# Geographic Variation in Diving Behaviour and Skin Isotope Ratios of Northern Bottlenose Whales, *Hyperoodon ampullatus*

**DOI:** 10.1002/ece3.73690

**Published:** 2026-05-27

**Authors:** Sascha K. Hooker, Eilidh Siegal, Tessa Plint, Laura J. Feyrer, Tomoko Narazaki, Kagari Aoki, Matt Bivins, Patrick J. O. Miller

**Affiliations:** ^1^ Sea Mammal Research Unit, Scottish Oceans Institute University of St Andrews St Andrews UK; ^2^ Scottish Marine Animal Stranding Scheme, School of Biodiversity, One Health, and Veterinary Medicine University of Glasgow Glasgow UK; ^3^ Dalhousie University Halifax Nova Scotia Canada; ^4^ Fisheries and Oceans Canada Maritimes Region Dartmouth Nova Scotia Canada; ^5^ Department of Environmental Bioscience Meijo University Nagoya Aichi Japan; ^6^ Atmosphere and Ocean Research Institute The University of Tokyo Chiba Japan

**Keywords:** anthropogenic impact, beaked whale, foraging, metapopulation, stable isotope, time budget

## Abstract

Geographic variation in the diving behaviour of oceanic mammals is a major component of their biodiversity with far‐reaching relevance for ecology and conservation. Here we compared high‐resolution multi‐sensor tag data from northern bottlenose whales in Jan Mayen, Norway (*n* = 15, 127.2 h) and the Gully, Scotian Shelf, Canada (*n* = 6, 45.8 h) to compare diving and foraging behaviours. *K*‐means clustering identified short‐shallow and long‐deep dive types in both locations but showed a propensity for mid‐depth dives in Jan Mayen. Foraging dives (defined by high roll variance corroborated by active clicking) included long‐deep dives (Jan Mayen = 967 ± 394 m SD, *n* = 26; Gully = 1247 ± 411 m, *n* = 14) but also these mid‐depth dives (537 ± 135 m, *n* = 105). Time budgets varied substantially between locations with whales in the Gully spending 15% less time foraging (*Z* = 2.1, *p* = 0.03) and twice as much time near‐surface resting (*Z* = −3.8, *p* < 0.001). Movement parameters further showed enigmatic gyrations during dives, more common in Jan Mayen. Skin biopsy stable isotopes revealed small regional differences, likely reflecting both diet and ecoregion (Jan Mayen *n* = 18: *δ* ^15^N 15.03‰ ± 0.35‰, Gully *n* = 41: *δ* ^15^N 15.41‰ ± 0.44‰). These population differences suggest unequal susceptibility to stressors and unequal likelihood of detection, with consequences for population management and abundance estimation.

## Graphical abstract

High‐resolution multi‐sensor tag data from northern bottlenose whale populations in the northeast and northwest Atlantic showed differences in resting and foraging time budgets and behaviour. Skin biopsy stable isotopes corroborated likely dietary differences. Variability between populations has far‐reaching relevance for ecology and conservation, with implications for animal susceptibility to stressors and our ability for detection and abundance estimation.

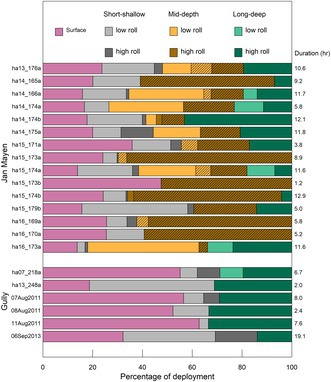

## 1 Introduction

Understanding inter‐population variation in behaviour is crucial for characterising phenotypic plasticity and geographic structure (Foster 1999), with consequences for understanding the impacts of anthropogenic pressures (Montague et al. 2013). This is critical for determining whether to manage species as single or separate stocks (Thomas and Stirling 1983) or for using different management approaches in different locations. Yet, despite its importance for conservation (Agrawal 2001), inter‐population variability in behaviour remains poorly understood, particularly for species whose behaviour cannot be directly observed.

Marine mammals live, move and forage in complex three‐dimensional underwater environments. Classifying and comparing dive types allows examination of inter‐population differences in behaviour (McHuron et al. 2018). Different dive types are often further classified by different behavioural functions such as foraging or travelling (Tennessen et al. 2019). Other factors (e.g., search strategy, predation risk, buoyancy control) can drive variation in movements during descent and ascent and from dives (Miller et al. 2016). Bio‐logging devices that contain triaxial accelerometers and magnetometers can reveal these multifaceted three‐dimensional movements.

Understanding how time is allocated during diving can also reveal behavioural differences (Bodkin et al. 2007). For example, time spent actively foraging versus descending, ascending and recovering at the surface can reveal intraspecific variation in proportion of time used in different dive phases.

Potential drivers of intra‐species variation are often thought to be associated with dietary differences, but prey composition and distribution are difficult to measure directly for deep‐diving species (often requiring specialist equipment such as dual frequency echosounder deployed via 600‐m depth‐capable Autonomous Underwater Vehicle, Southall et al. 2019). Stable isotope analyses can instead provide a broad comparator of diet eaten during the past weeks–months (depending on tissue used). The carbon and nitrogen isotopes found in animal tissues reflect the food resources that have been assimilated, with predictable enrichment in heavy isotopes (^13^C and ^15^N) with each associated increase in trophic level or with specific habitat use (Hobson et al. 1994; Espinasse et al. 2022).

Beaked whale populations are particularly vulnerable to anthropogenic threats (Feyrer, Stanistreet, and Moors‐Murphy 2024). However, despite recent increases in research effort (Hooker et al. 2019), we still know little about intraspecific variation in behaviour. Studies have suggested that they perform deep dives consistent with foraging on deep‐water squid and fish, with dives typically falling into two categories: short duration shallow non‐foraging dives and long duration deep foraging dives (Hooker and Baird 1999; Tyack et al. 2006; Shearer et al. 2019). Goose‐beaked whales ( *Ziphius cavirostris* ) and dense‐beaked whales ( *Mesoplodon densirostris* ) show regional differences in depth, duration and inter‐dive intervals (Shearer et al. 2019; Coates et al. 2024; Barlow et al. 2025), but differences in prey that might have caused this variation were not examined and population variation has not been explored for other beaked whale species.

**FIGURE 1:**
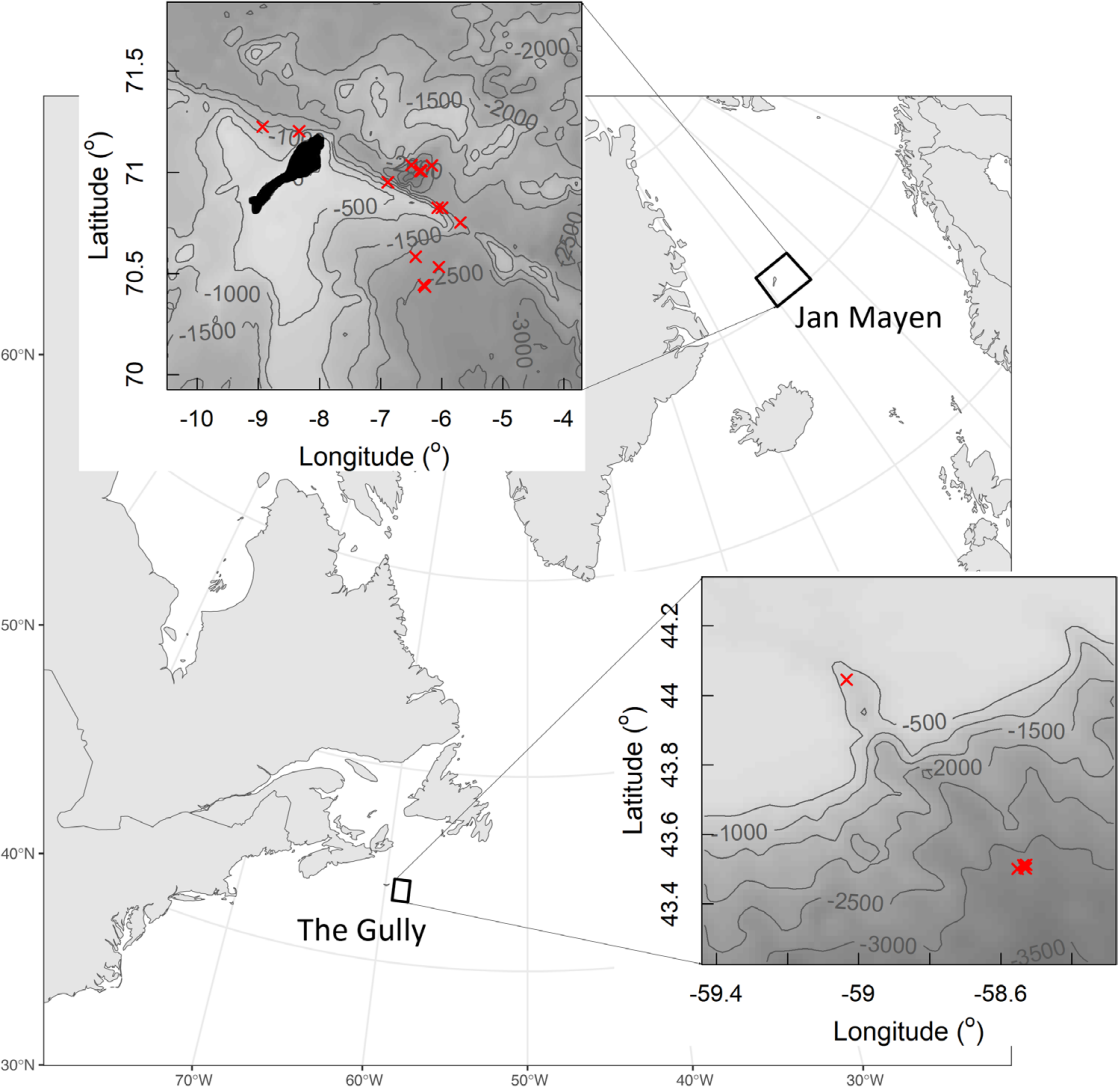
Map of the North Atlantic showing the location of the two study sites: The Gully, Canada, and Jan Mayen, Norway. The location of movement‐recording tags deployed on northern bottlenose whales are shown as red crosses (Gully *n* = 6, Jan Mayen *n* = 15). Depths are given in metres.

Northern bottlenose whales ( *Hyperoodon ampullatus* ) are distributed across the northern North Atlantic. Study effort has primarily focused on the western North Atlantic Scotian Shelf population centred around the Gully and neighbouring submarine canyons (Hooker et al. 2019), but recent work in the eastern North Atlantic has included a submarine canyon system around Jan Mayen (Figure 1; Miller et al. 2015; Neubarth et al. 2025). Northern bottlenose whales were thought to consist of a single largely undifferentiated population (Whitehead and Hooker 2012), but recent assessment has suggested genetic subdivision distinguishing the Scotian Shelf, western North Atlantic and Jan Mayen regions (de Greef et al. 2022). Bottlenose whales in the eastern Atlantic undertake seasonal migrations whereas those on the Scotian Shelf likely do not (Whitehead and Hooker 2012). Lower body density (indicating larger lipid stores) has been found for northern bottlenose whales in Jan Mayen compared to the Scotian Shelf, possibly related to energy storage prior to migrations (Miller et al. 2016). Whales in both locations typically perform deep‐water foraging dives during which they echolocate to detect and capture their prey (Hooker and Baird 1999; Miller et al. 2015). Beaked whales usually remain silent in the upper 400 m of the water column for acoustic crypsis (Aguilar de Soto et al. 2012), but bottlenose whales have been recorded to perform clicks while near the surface (Hooker and Whitehead 2002; Miller et al. 2015), and some of their clicks may serve a social function (Haas et al. 2025).

Using data from biologging devices, this study investigates geographic variation in diving behaviour and skin isotope ratios of northern bottlenose whales in the north‐eastern Atlantic Jan Mayen population versus the north‐western Atlantic Scotian Shelf population. Three key components of diving behaviour are investigated: (1) dive types and foraging depth; (2) time budgets; and (3) kinematic movements within and between dives. Stable isotope assessment of whale samples from the two locations is used to help interpret results.

## 2 Materials and Methods

### 2.1 Dataset

Fieldwork was associated with various projects and took place around Jan Mayen, Norway, and the Gully, Scotian Shelf, Canada (Figure 1). Tagging of northern bottlenose whales took place in Jan Mayen (*n* = 15, 127.2 h) using sailing (2014: *Prolific*; 2015 and 2016: *Donna Wood*) and research (2013: *HU Sverdrup II*) vessels, and in the Gully using the F/V *On a Mission* (*n* = 6, 45.8 h) (Table 2). Biopsying took place from these vessels in Jan Mayen and sailing vessel *Balaena* on the Scotian Shelf.

Animal‐attached loggers consisted of DTag‐2, DTag‐3, Mixed‐DTags (M‐DTag, consisting of a DTag‐3, Fastloc‐GPS logger and SPOT transmitter) or 3MPD3GT (Little Leonardo, Japan) loggers. DTags sampled audio at 192–240 kHz and pressure, 3‐axis accelerometer (±2 *g*) and 3‐axis magnetometer at 50 Hz (DTag‐2) or 250 Hz (DTag‐3 and M‐DTag, down‐sampled using low‐pass anti‐alias filter to 50 Hz). 3MPD3GT loggers sampled 3‐axis (±3 *g*) acceleration at 32 Hz, plus pressure, 3‐axis magnetism and speed (from a paddlewheel) at 1 Hz. The 3MPD3GT loggers attach to whales via a single suction cup, which contrasts to the more rigid four suction‐cup attachment of DTags. 3MPD3GT loggers do not contain audio sensors. No audio data were available for the Gully as Gully DTag sensors either malfunctioned or no focal sounds were recorded.

Four Jan Mayen deployments included controlled sonar exposures (Table 2), for which only baseline data prior to these exposures were included here. To reduce potential effects of the tagging process on behaviour, the first 20 min of each deployment was excluded from analyses.

As tags were attached to whales at various orientations, sensor data were calibrated and converted from tag‐frame to whale‐frame axes using established methods (Johnson and Tyack 2003; Miller, Johnson, Tyack, and Terray 2004). Kinematics are generally described as body orientation changes, consisting of pitch, roll and heading determined from *prhpredictor* in the DTag toolbox processed with MATLAB (SoundTag 2016).

In both locations, deep dives with high roll variance in the bottom phase consistently commenced 20 min after tag attachment so we assume that behaviour returned to ‘normal’ after this time. The bottom phase was defined using pitch, as in Miller, Johnson, Tyack, and Terray (2004), where the bottom phase is the time from the end of the descent (the animal's pitch first exceeds 0°) until the start of the ascent (the animal's pitch was last below 0°).

Biopsy samples of skin and outer blubber were collected using the ARTS launching system (Kleivane et al. 2022) or crossbow from 5 to 15 m distance (Scotian Shelf, 1997–2016, Jan Mayen, 2014–2016). Samples were stored frozen until analysis. Genetic analyses provided sex identification (Gowans, Dalebout, et al. 2000).

### 2.2 Dive Classification and Function

Submergences deeper than 40 m were considered a ‘dive’ (Hooker and Baird 1999), and dive phases were defined via pitch (negative for descent, mixed for bottom, positive for ascent) (Miller, Johnson, Tyack, and Terray 2004). Dive types were identified for Jan Mayen and Gully datasets separately via *K*‐means, based on dive duration (s), maximum dive depth (m), ascent and descent rate (ms^−1^), using the silhouette coefficient to determine the appropriate number of clusters, *K* (Arbelaitz et al. 2013). Silhouette widths range from −1 (dive has no relationship to dives within its cluster) to +1 (dive is an exact match to its cluster). The silhouette coefficient (SC) is overall average silhouette width (> 0.5 indicates a reasonable grouping structure, and > 0.7 indicates a strong grouping structure), and the highest SC determines the optimal number of clusters (dive types) for each location (Rousseeuw 1987). To maintain comparability with previous studies, the cluster analysis was not scaled. It will therefore have relied more heavily on depth and duration than on ascent and descent rate. Silhouette values were estimated using the *cluster* and *factoextra* R packages. To ensure that sample size did not unduly affect the number of dive type clusters identified, six deployments (i.e., similar dataset size to the Gully) were randomly selected without replacement from the Jan Mayen dataset and SC was recalculated. This process was repeated 30 times. The Gully dataset was similarly examined with the incorporation of the previously published Hooker and Baird (1999) dataset.

Comparisons across dive types and locations were made for clustering parameters (depth, duration, ascent and descent rates) and additional foraging parameters measured in the bottom phase of dives: (1) roll variance; (2) heading variance; (3) jerk coefficient of variation (jerk CV); and (4) percentage of dive spent in bottom phase. Roll and heading variance were estimated as circular variance (continuous variables bound between 0 and 1; Isojunno et al. 2017). Values closer to 0 represent low sinuosity (few angle changes), while values closer to 1 represent high sinuosity (numerous angle changes). Jerk CV was the ratio of standard deviation to the mean. This is robust to variation in determination of jerk amplitude caused by slight differences between the tag sampling rate (Dtag 50 Hz and 3MPD3GT 32 Hz).

Dives were classified into foraging and non‐foraging dives. Foraging dives have previously been defined by the presence of regular clicking (Miller et al. 2015), but since this was available only for the Jan Mayen dataset, we examined roll variance in the bottom phase as an alternative indicator applied across all dives. High roll angles are often associated with the prey‐capture attempts of odontocetes (e.g., Tennessen et al. 2019). An optimal roll variance threshold was estimated, above which high roll variance dives were considered ‘foraging dives’ and below which low roll variance dives were considered ‘non‐foraging dives’.

To test the hypothesis that Gully and Jan Mayen animals vary in foraging behaviour, we compared the following foraging dive characteristics between locations: heading variance, jerk CV, proportion of dive spent in the bottom phase (bottom phase duration/dive duration) and proportion of time spent in bottom phase (bottom phase duration/(dive duration + post‐dive time until next foraging dive)). The last foraging dive of each deployment was excluded since post‐dive time was not available. With the characteristics of foraging dives (Gully *n* = 26, Jan Mayen *n* = 141) as the response and location as the fixed effect predictor, we used mixed models (with whale identity as a random effect of intercept) to account for autocorrelation between dives of the same deployment. Depending on the response variable, two types of mixed model were implemented. Beta mixed models (BMMs, fit under a maximum likelihood approach and a logit link function via the *GLMMadaptive* R Package) quantified variation in diving efficiency, percentage of time spent in the bottom phase and heading variance in terms of whether there was a difference in location for each of the three models. A linear mixed model (LMM, fit under a restricted maximum likelihood approach and identity link function via the *lme4* R package, with checks for outliers and normality) assessed variation in jerk CV, assuming a Gaussian error distribution. Due to the small Gully sample size, the significance of the LMM fixed effects was evaluated via Kenward‐Roger approximation (via the *pbktest* R package and using restricted maximum likelihood as required for this, Kenward and Roger 1997) to provide more optimal Type 1 error rates (Luke 2017). Alpha levels of 0.05 and 0.10 represented strong and moderate support respectively.

### 2.3 Time Budgets

To test the hypothesis that Gully and Jan Mayen animals varied in how whales budgeted their time, we compared the percentage of time spent: (1) at the surface (in‐between dives deeper than 40 m); (2) in foraging dives; and (3) in the bottom phase of foraging dives. We used time budgets of deployments (Gully *n* = 6, Jan Mayen *n* = 15) as the response variable and location as a fixed effect in beta regression models (using the *betareg* R Package) with a small sample size correction (Simas et al. 2010) separately for each of these proportions.

### 2.4 Three‐Dimensional Movements

Heading tortuosity was estimated as tortuosity index (TI): the ratio of net horizontal distance to the total horizontal distance covered given a constant heading and speed (Tyack et al. 2006); TI = 0 indicating straight‐line movement, TI = 1 indicating perfectly circular movement. Changes in orientation (roll variance and heading tortuosity) were estimated for 50‐m depth bands during ascent and descent, and while whales were at the surface (time between dives > 40 m). Dive profiles were plotted in three dimensions colour coded for pitch and roll, using pseudo‐tracks estimated via dead‐reckoning from body pointing angle and assuming a constant speed of 2 m s^−1^ (TagTools; Johnson et al. 2009). While these help with visual interpretation, it should be noted that they incur additive dead‐reckoning errors as the track progresses further from surface locations.

### 2.5 Stable Isotope Analyses

Analysis of skin samples from the Scotian Shelf (1997) has previously been described (Hooker et al. 2001). Skin samples from the Scotian Shelf (2013, 2015, 2016) and Jan Mayen (2014, 2016) were lipid extracted following a modified Bligh and Dyer (1959) method (three rinses of 60 min each in 2:1 chloroform: methanol) prior to drying at 60°C.

Stable isotope analyses of Jan Mayen skin samples were measured using Elementar Vario Isotope Select elemental analyser interfaced with an Isoprime 100 continuous flow isotope ratio mass spectrometer, using helium as the carrier gas at the Stable Isotope Mass Spectrometry Laboratory (SIRMS lab) at the University of Southampton. Skin sample duplicates (*n* = 3) were included. Skin samples were normalised using international standards USGS‐40 (L‐glutamic acid: 1SD *δ* ^13^C = 0.2‰, accepted *δ* ^13^C = −26.39‰ ± 0.04‰; 1SD *δ* ^15^N = 0.2‰, accepted *δ* ^15^N = −4.52‰ ± 0.06‰) and USGS‐41 (L‐glutamic acid; 1SD *δ* ^13^C = 0.2‰, accepted *δ* ^13^C = +36.63‰ ± 0.05‰; 1SD *δ* ^15^N = 0.2‰, accepted *δ* ^15^N = +47.57‰ ± 0.11‰). The internal laboratory reference material ‘Atlantic cod muscle’ was used to determine analytical precision (1SD *δ* ^13^C = 0.1‰, accepted *δ* ^13^C = −19.31‰ ± 0.11‰; 1SD *δ* ^15^N = 0.1‰, accepted *δ* ^15^N = +11.41‰ ± 0.19‰), while acetanilide was used to determine carbon and nitrogen elemental (%) composition. Analytical error was 0.2‰ for both *δ* ^13^C and *δ* ^15^N. Sample duplicates (*n* = 3) differed by 0.15‰ and < 0.1‰ for *δ* ^13^C and *δ* ^15^N, respectively.

Stable isotope analyses of Scotian Shelf skin samples were measured using a Delta V Advantage ThermoScientific Continuous Flow Mass Spectrometer (Thermo Scientific, Bremen, Germany) coupled to a 4010 Elemental Combustion System (Costech Instruments, Valencia, CA, USA) in the GLIER Chemical Tracers Laboratory at the University of Windsor. Skin samples were normalised using international standards USGS‐40 (*n* = 10; L‐glutamic acid: 1SD *δ* ^13^C = 0.2‰, accepted = −26.39‰ ± 0.04‰; 1SD *δ* ^15^N = 0.0‰, accepted = −4.52‰ ± 0.06‰), USGS‐41 (*n* = 10; L‐glutamic acid: 1SD *δ* ^13^C = 0.11‰, accepted *δ* ^13^C = +36.63‰ ± 0.05‰; 1SD *δ* ^15^N = 0.02‰, accepted *δ* ^15^N = +47.57‰ ± 0.11‰), IAEA‐N‐1 (*n* = 10; ammonium sulphate: 1SD *δ* ^15^N = 0.01‰, accepted *δ* ^15^N = +0.43‰), and IAEA‐CH‐6 (*n* = 10; sucrose: 1SD *δ* ^13^C = 0.15‰, accepted *δ* ^13^C = −10.45‰). Four additional standards (NIST 1577c [bovine liver], tilapia muscle, USGS‐40 and Urea; *n* = 8 each) were used to determine analytical precision (< 0.14‰) for both *δ* ^13^C and *δ* ^15^N.

All carbon and nitrogen isotope results are reported in *δ*‐notation in per mil (‰) relative to international standards calibrated to VPDB and AIR, respectively. *δ* ^13^C was Suess and Laws effect corrected to 2016 using the SuessR package in R for comparisons across collection years (region: subarctic North Atlantic; Clark et al. 2021). Suess and Laws effect corrections account for the progressive ^13^C‐depletion of atmospheric CO_2_ following anthropogenic burning of isotopically light fossil fuels since 1850, and the impact of increasing ocean temperature on marine primary productivity (Sonnerup et al. 1999). The combined magnitude of Suess and Laws effect correction over an 8‐year period exceeds that of acceptable isotope ratio mass spectrometer instrument error (±0.2‰). Results were corrected to 2016 to render *δ* ^13^C data comparable between biopsy collection years. Isotopic niches were determined using the SIBER package (Stable Isotope Bayesian Ellipses in R; Jackson et al. 2011), using Bayesian multivariate normal distributions, calculating core (40% convex hull) and total (100% convex hull) isotopic niches.

A correction based on differences between locations was applied based on spatial variation in carbon and nitrogen isoscape across the North Atlantic (Smith et al. 2021; Espinasse et al. 2022). Mean zooplankton sample *δ* ^13^C and *δ* ^15^N differ between Jan Mayen (situated within ‘Ecoregion 3’ of the North Atlantic; mean *δ* ^13^C = −23.61‰ ± 0.33‰, mean *δ* ^15^N = +6.47‰ ± 0.46‰) and the Scotian Shelf (situated within ‘Ecoregion 1’; mean *δ* ^13^C = −21.89‰ ± 0.39‰; *δ* ^15^N = +5.28‰ ± 0.95‰) (Espinasse et al. 2022). Baseline whale carbon isoscape values in Jan Mayen (Ecoregion 3) were therefore expected to be approximately +1.7‰ higher than those in the Scotian Shelf (Ecoregion 1), and baseline nitrogen isoscapes values approximately −1.2‰ lower than the Scotian Shelf.

## 3 Results

### 3.1 Dive Classification and Function

Across the two locations 21 tag deployments were recorded, with average (±SD) durations of 8.5 h (±3.7, *n* = 15) in Jan Mayen and 7.6 h (±6.2, *n* = 6) in the Gully (Table 1). Gully deployments were on average 1 h shorter; the mean number of dives identified per deployment (12.7 ± 15.2) was roughly half that for Jan Mayen deployments (24.3 ± 11.0), resulting in 76 dives in the Gully and 364 in Jan Mayen.

**TABLE 1:** Details of digital movement‐recording tags deployed on northern bottlenose whales off Jan Mayen, Norway (*n* = 15), and in the Gully, Canada (*n* = 6).

Tag ID	Location	Tag type	Date and time	Duration (hr)	Descent roll gyrations	Ascent heading gyrations
ha13_176a^c^	Jan Mayen	DTag‐2	25/06/2013 01:09	10.6	8	4
ha14_165a	Jan Mayen	DTag‐2	14/06/2014 18:03	9.2	7	2
ha14_166a	Jan Mayen	DTag‐2	15/06/2014 08:22	11.7	6	4
ha14_174a	Jan Mayen	DTag‐2	23/06/2014 07:30	5.8	2	5
ha14_174b	Jan Mayen	DTag‐2	23/06/2014 09:18	12.1	5	1
ha14_175a	Jan Mayen	DTag‐2	24/06/2014 14:09	11.8	1	7
ha15_171a^c^	Jan Mayen	DTag‐2	20/06/2015 11:27	3.8	1	1
ha15_173a	Jan Mayen	DTag‐2	22/06/2015 13:52	8.9	2	3
ha15_174a	Jan Mayen	DTag‐2	23/06/2015 07:29	11.6	3	3
ha15_173b	Jan Mayen	DTag‐3	22/06/2015 17:27	1.2	0	0
ha15_174b	Jan Mayen	DTag‐3	23/06/2015 08:34	12.9	2	1
ha15_179b^c^	Jan Mayen	DTag‐3	28/06/2015 21:49	5.0	0	0
ha16_169a	Jan Mayen	M‐DTag	17/06/2016 22:44	5.8	2	1
ha16_170a^c^	Jan Mayen	M‐DTag	18/06/2016 07:06	5.2	1	0
ha16_173a	Jan Mayen	M‐DTag	21/06/2016 09:01	11.6	2	0
ha07_218a^a^	Gully	DTag‐2	06/08/2007 10:54	6.7	1	0
ha13_248a^b^	Gully	DTag‐3	05/09/2013 15:04	2.0	0	0
07Aug2011	Gully	3MPD3GT	07/08/2011 09:30	8.0	0	0
08Aug2011	Gully	3MPD3GT	08/08/2011 09:19	2.4	0	0
11Aug2011	Gully	3MPD3GT	11/08/2011 07:44	7.6	1	0
06Sep2013	Gully	3MPD3GT	06/09/2013 15:08	19.1	0	4

*Note:* Tag‐on date and time are given in local time. Number of circular gyrations is number of observed incidences where an animal undertook a minimum of two continuous rotations in (1) roll during the descent phase and (2) heading during the ascent phase from mid‐depth and long‐deep dives.

^a^ Sound recorded but no focal clicks recorded.

^b^ Audio sensors did not function.

^c^ Pre‐exposure deployment (prior to controlled sonar exposure).

**FIGURE 2:**
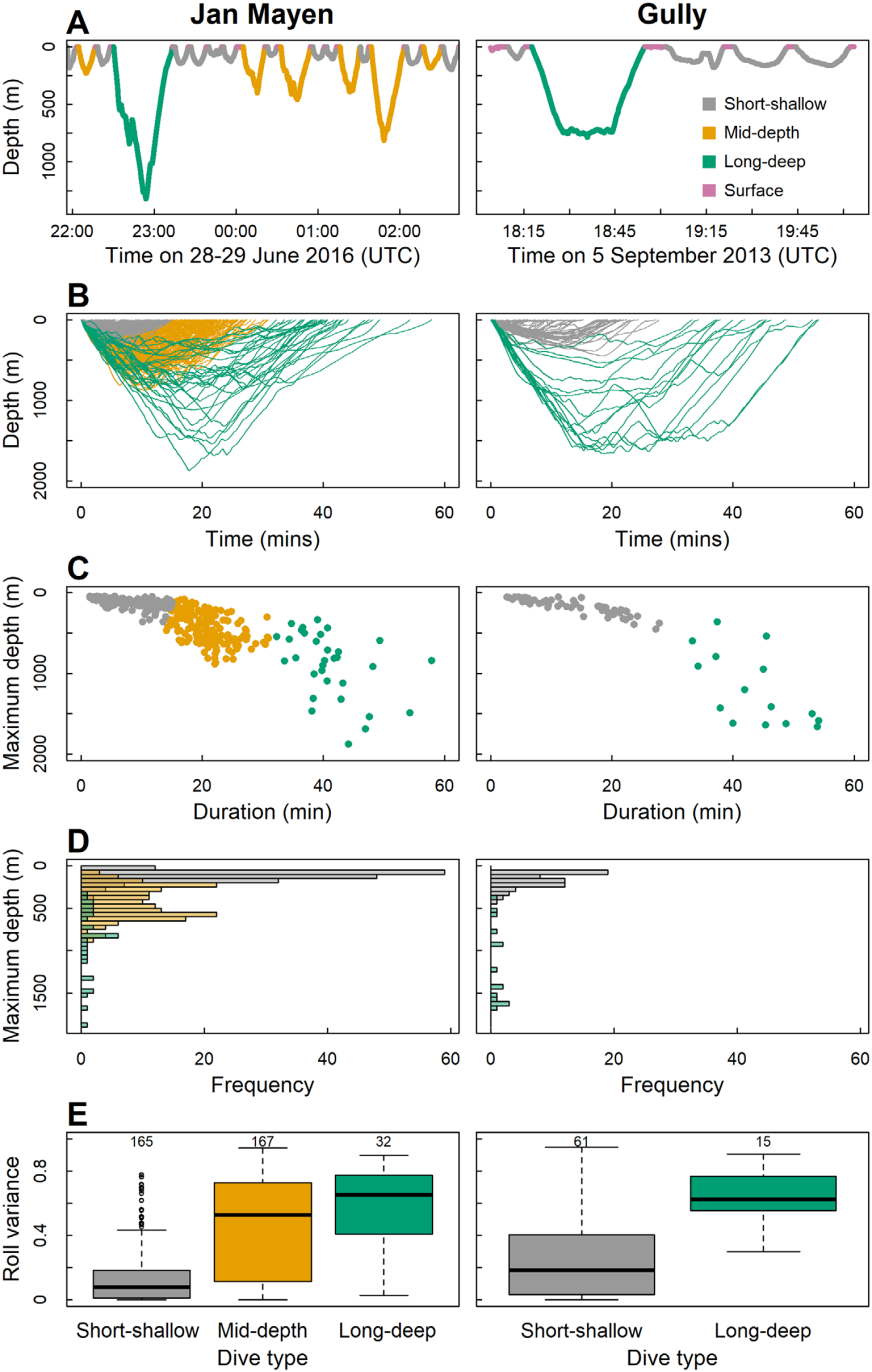
Dive types (identified by *K*‐means clustering) of northern bottlenose whales in Jan Mayen, Norway (left), and the Gully, Canada (right). Dive type (grey = short‐shallow, orange = mid‐depth, green = long‐deep, pink = near surface). (A) Depth time series for ha15_179b (Jan Mayen) and ha13_248a (Gully). (B) Time‐depth profiles of all dives. (C) Maximum depth and duration of dives. (D) Overlaid histograms of maximum dive depth. (E) Box‐plots to show range of roll variance within these dive types.

**FIGURE A1:**
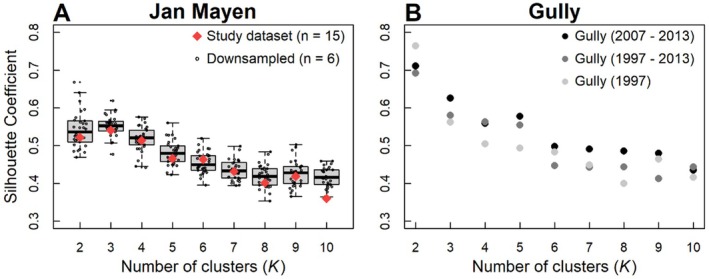
Classification of dives for northern bottlenose whales off Jan Mayen, Norway (left), and the Gully, Canada (right), using the silhouette coefficient (SC) and *K*‐means clustering. (A) The SC at different numbers of clusters (*K*), shown for all 15 deployments around Jan Mayen (red diamonds) and for 30 iterations randomly subsampling 6 deployments (boxplots and black circles). (B) SC shown for Gully dataset (six deployments in 2007–2013), the initial Gully dataset (two deployments in 1997; Hooker and Baird 1999) and both datasets combined.

Using dive duration, depth, ascent and descent rate of dives, the silhouette coefficient (SC) suggested three dive types in Jan Mayen (short‐shallow, mid‐depth and long‐deep; Figure 2), robust to reduced sample size and with reasonable group structure (SC = 0.54, Appendix 1: Figure A1). In the Gully, the highest SC value (0.71) was for two clusters (short‐shallow and long‐deep dives; Figure 2), and indicated a strong group structure that was robust to inclusion of the Gully 1997 dataset (Appendix 1: Figure A1). Adding bottom phase duration did not improve cluster fit (Jan Mayen *K* = 3: SC = 0.48; Gully *K* = 2: SC = 0.69). Short‐shallow dives were identified in both locations, but these were longer, deeper and had slower ascent and descent rates in the Gully (Table 2). Long‐deep dives were deeper in the Gully, but similar in duration in both locations. Mid‐depth dives were the most numerous dive type (46% of dives) in Jan Mayen, but this class was not identified in the Gully, and dives to this depth were rare (Table 2, Figure 2).

**TABLE 2:** Dive metrics used in *K*‐means clusters to identify dive types of northern bottlenose whales.

Parameter	Dive type	Location	
		Jan Mayen M (±SD)	Gully M (±SD)
Depth (m)	Short‐shallow	121.6 (61.9)	171.6 (96.0)
	Mid‐depth	430.4 (189.6)	
	Long‐deep	888.8 (407.4)	1188.0 (456.8)
Duration (min)	Short‐shallow	8.2 (4.0)	13.9 (7.2)
	Mid‐depth	20.5 (3.9)	
	Long‐deep	41.1 (5.7)	43.6 (6.9)
Ascent rate (ms^−1^)	Short‐shallow	0.7 (0.3)	0.5 (0.2)
	Mid‐depth	1.0 (0.4)	
	Long‐deep	0.8 (0.3)	1.1 (0.4)
Descent rate (ms^−1^)	Short‐shallow	0.9 (0.5)	0.6 (0.3)
	Mid‐depth	1.3 (0.4)	
	Long‐deep	1.4 (0.4)	1.6 (0.3)

*Note:* Mean values (M) and standard deviation (SD) shown for short‐shallow (Jan Mayen *n* = 165, Gully *n* = 61), mid‐depth (Jan Mayen *n* = 167) and long‐deep dives (Jan Mayen *n* = 32, Gully *n* = 15).

**FIGURE 3:**
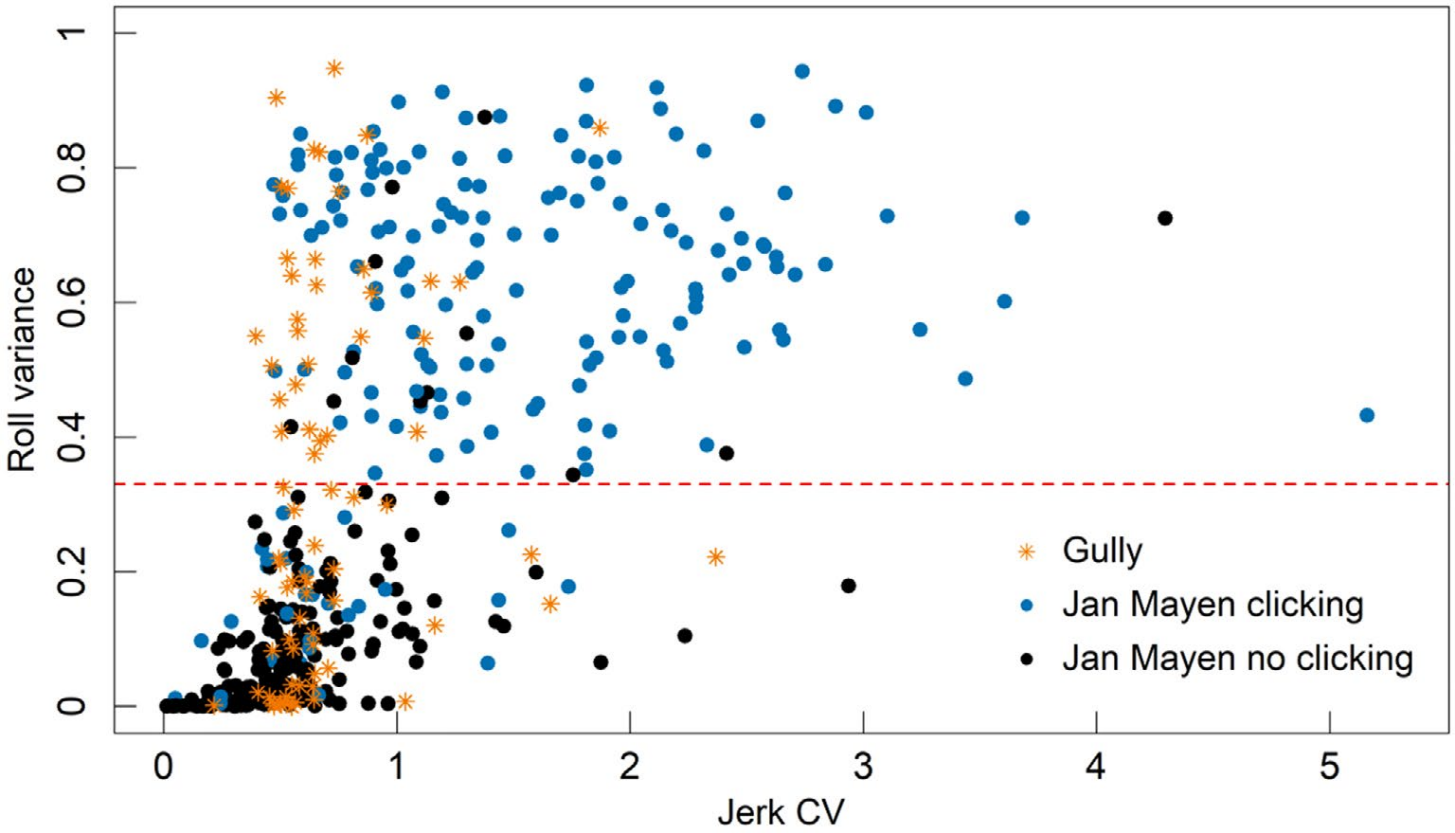
Comparison of roll variance, jerk CV and presence of regular clicking as indicators of foraging among Jan Mayen records compared to dives from Gully records (without click information). Plot shows Jerk CV and roll variance in the bottom phase. The optimal roll‐variance threshold for identifying foraging dives is marked (dashed red line). Blue circles represent presence (*n* = 172) and black circles represent absence (*n* = 192) of regular clicking during dives around Jan Mayen. Dives in the Gully are shown as orange stars (*n* = 76).

**FIGURE 4:**
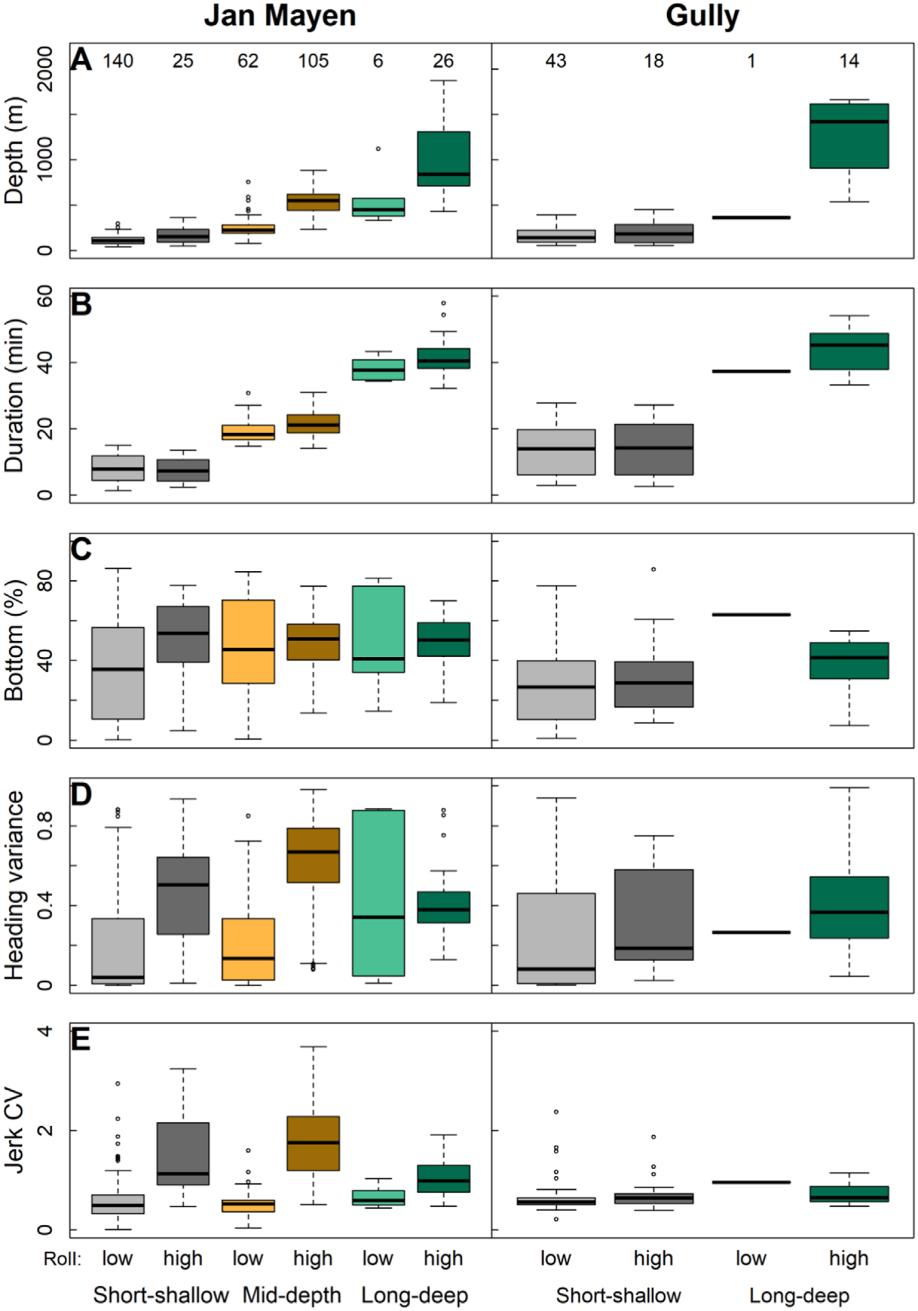
Differences between parameters for dive types subdivided by high/low roll variance in Jan Mayen, Norway (left), and the Gully, Canada (right). Dive types (green = long‐deep, orange = mid‐depth, grey = short‐shallow) are divided into high and low roll variance (threshold = 0.33). (A) Maximum dive depth. (B) Dive duration. (C) Percentage of dive spent in the bottom phase. (D) Heading variance in the bottom phase. (E) Jerk coefficient of variation (CV) in the bottom phase.

**FIGURE A2:**
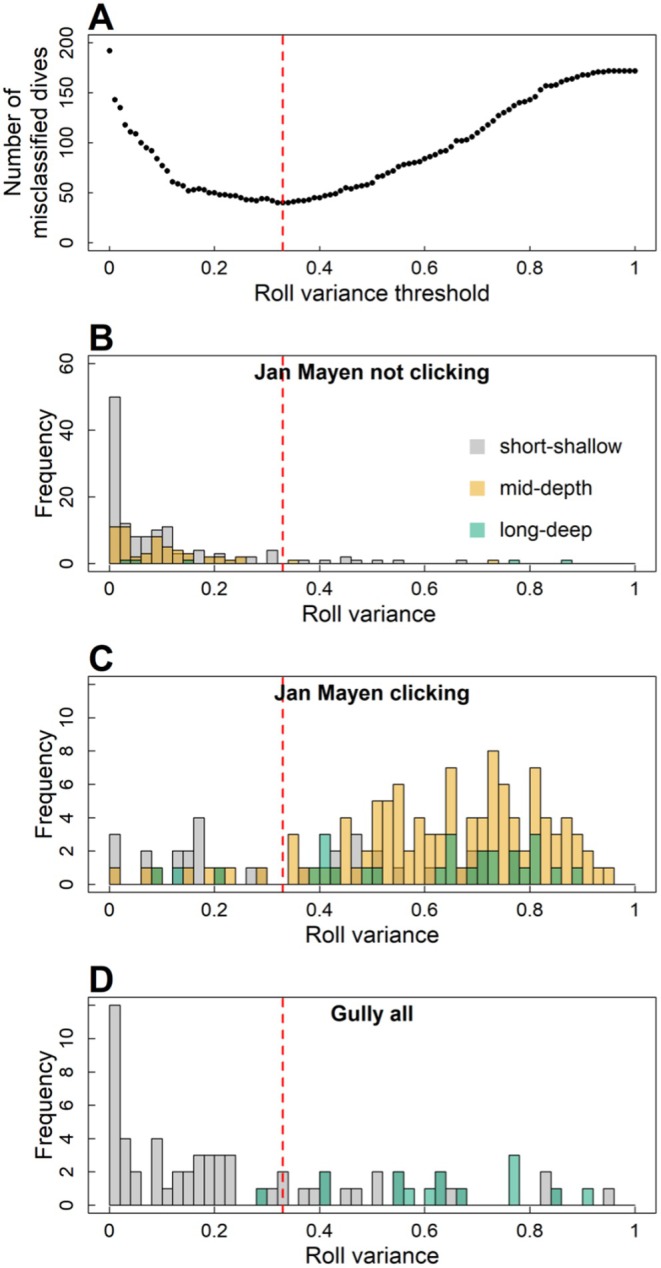
Classification of dives using an optimal roll‐variance threshold. Roll variance is the circular variance of roll during the bottom phase of dives. (A) Scatterplot shows number of mismatched Jan Mayen dives using different roll‐variance thresholds, with minima at optimal threshold (0.33, dashed red line). (B–D) Frequency histograms show proportion of dives according to roll variance. (B) Jan Mayen dives without regular echolocation. (C) Jan Mayen dives with regular echolocation clicking. (D) Gully dives (clicking information not available). Colour represents dive type (green = long‐deep, orange = mid‐depth, grey = short‐shallow).

Comparing bottom phase roll variance with acoustic clicking as an indicator of foraging dives, a bottom phase roll variance > 0.33 minimised the number of different classifications (Appendix 2: Figure A2), with 28 clicking dives in Jan Mayen (17 short‐shallow, 8 mid‐depth and 3 long‐deep) classed as ‘non‐foraging’, and 12 non‐clicking dives (8 short‐shallow, 2 mid‐depth and 2 long‐deep) classed as ‘foraging’. Using this high roll variance classifier, foraging in both locations had high jerk CV (Figure 3), high heading variance, and high percentage of time in the bottom phase (Figure 4). Lack of clicking among some of these high roll variance dives suggested that some foraging dives may be silent (Figure 3).

Foraging dives were predominantly long‐deep and mid‐depth dives (Figure 4). Long‐deep foraging dives in the Gully were deeper (IQR = 918–1609 m) than in Jan Mayen (IQR = 715–1255 m), and only four foraging dives in the Gully were to depths of 300–600 m. In contrast, in Jan Mayen foraging dives consistently occurred at mid‐depths (IQR = 441–620 m) and were the most prevalent foraging dive type.

At both sites, some possible shallow foraging dives occurred: 18 of 61 short‐shallow dives in the Gully (29.5%) and 25 of 165 short‐shallow dives (15.2%) in Jan Mayen had high roll variance (Figure 4). These dives were to depths of 170 ± 95 m in Jan Mayen and 197 ± 109 m in the Gully. In Jan Mayen, these dives had distinctly higher jerk CV, heading variance and percentage of time in the bottom phase than the low roll variance equivalents (Figure 4). However, in the Gully, although short‐shallow high roll variance dives had slightly higher heading variance, both jerk CV and percentage of time in the bottom phase were comparable to low roll variance dives (Figure 4).

Heading variance, jerk CV and the percentage of time spent in the bottom phase of foraging dives were all lower in the Gully than in Jan Mayen (Table 3, Figure 4). Interestingly, these differences were largely driven by mid‐depth foraging dives in Jan Mayen. In fact, long‐deep foraging dives had similar heading variance in both locations (Jan Mayen: 0.42 ± 0.19; Gully: 0.42 ± 0.26), while the heading variance of mid‐depth foraging dives was greater (0.61 ± 0.24). Jerk CV during mid‐depth foraging dives in Jan Mayen (1.76 ± 0.75) was also higher than that during long‐deep foraging dives (1.00 ± 0.35).

**TABLE 3:** Time budgets and foraging dive characteristics for northern bottlenose whales in Jan Mayen, Norway compared to the Gully, Canada.

Parameter	Jan Mayen M (SD)	Gully M (SD)	Model	β ± SE	Test statistic	*p*
Time budget						
% surface	22.7 (9.0)	46.2 (17.0)	BM	1.0 ± 0.3	Z = 3.8	**< 0.001**
% foraging dives	47.0 (14.9)	32.2 (2.4)	BM	−0.6 ± 0.3	Z = −2.1	0.03
% bottom phase foraging	22.9 (7.5)	12.0 (1.9)	BM	−0.7 ± 0.2	Z = −3.1	**0.002**
Foraging dive characteristics						
Diving efficiency (%)	25.9 (17.5)	12.6 (18.9)	BMM	−0.5 ± 0.2	Z = −2.2	0.03
Bottom phase (%)	49.5 (15.0)	32.1 (17.6)	BMM	−0.8 ± 0.2	Z = −5.0	**< 0.001**
Heading variance	0.6 (0.3)	0.3 (0.2)	BMM	−0.9 ± 0.2	Z = −3.9	**< 0.001**
Jerk CV	1.7 (0.8)	0.7 (0.3)	LMM	−0.9 ± 0.2	F = 23.6	**< 0.001**

*Note:* Time budgets are the proportion of deployment duration (expressed as the percentage of deployments; Gully *n* = 6, Jan Mayen *n* = 15) spent within 40 m of the surface, in foraging, and in the bottom phase of these dives. Time budgets were compared using beta models (BM) with a small sample size bias correction. Foraging dive characteristics (Gully *n* = 26, Jan Mayen *n* = 141) were compared using beta mixed models (BMM) or a linear mixed model (LMM), with location as a fixed effect and deployment as a random effect of intercept. Numerator degrees of freedom and denominator degrees of freedom for *F* statistic (LMM) are 1 and 159.6 respectively. Test results include coefficient estimates ± standard error (β ± SE), test statistics and *p* values. Given seven significance tests, a Bonferroni adjustment to significance threshold (0.05/7 = 0.007) was applied, and *p* values assessed as significant at the corrected threshold are shown in bold.

### 3.2 Time Budgets

**FIGURE 5:**
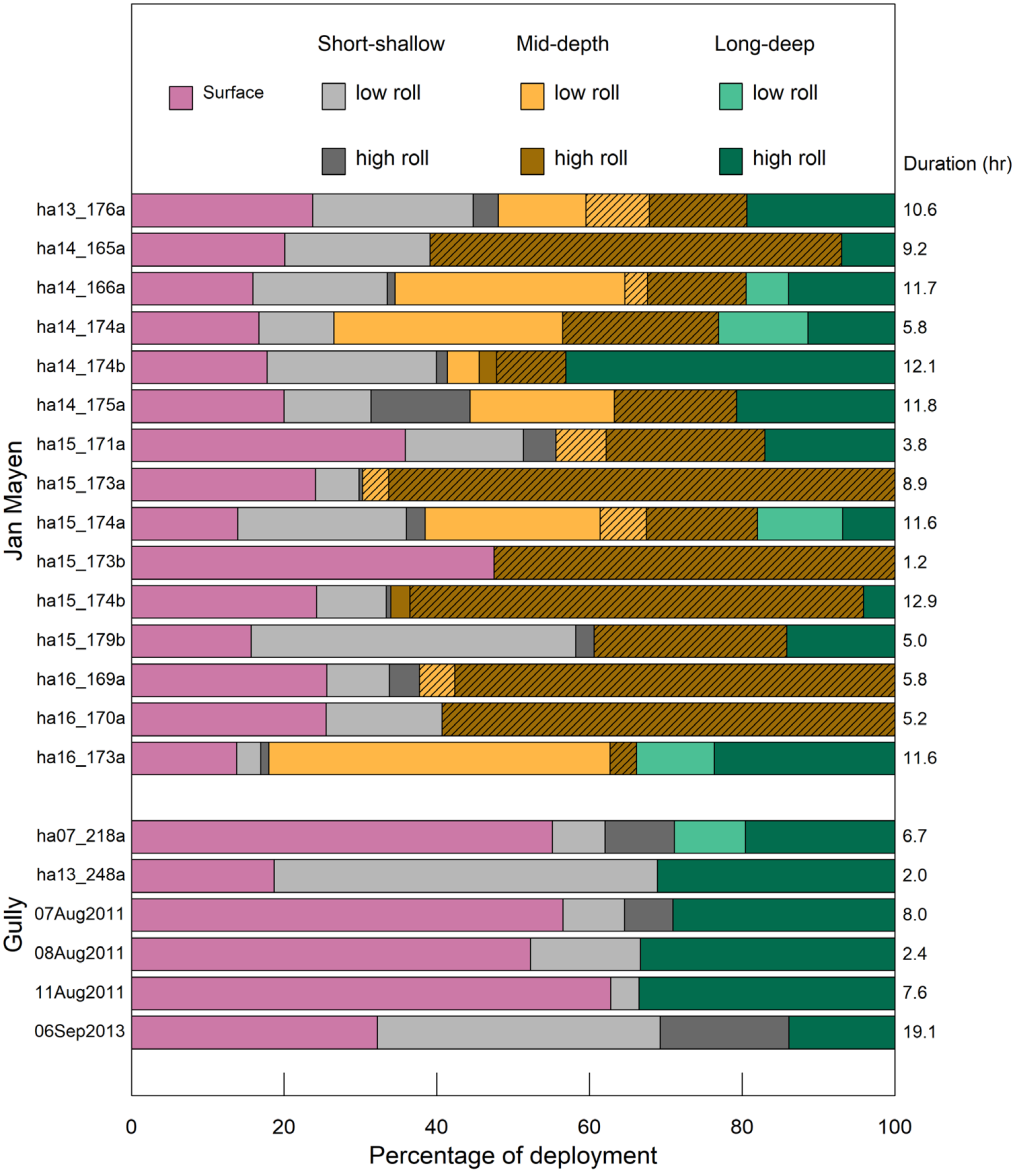
Individual time budgets (% time) at surface and in different dive types (*K*‐means clusters), subdivided by roll variance. Hatching shows dives (in Jan Mayen) with acoustic clicking. Time at the surface includes all excursions < 40 m deep.

There was strong support that the percentage of time spent near surface (i.e., between dives > 40 m) varied by location (Table 3, Figure 5). Whales in the Gully consistently spent more time near surface (Gully IQR = 37%–56%; Jan Mayen IQR = 16%–25%), with four of six Gully whales spending over half of their time near surface.

In Jan Mayen, whales spent a higher proportion of time (47% ± 15% of the deployment) in foraging dives, although this varied more (range = 24%–67%) than in the Gully (range = 29%–35%; Table 3, Figure 5). Whales in Jan Mayen also spent relatively more time (> 1.8 more) in the bottom phase of foraging dives relative to whales in the Gully (Table 3).

Of the foraging dives, whales in Jan Mayen spent most time in mid‐depth dives (33% ± 23%), compared to long‐deep dives (12% ± 12%) or short‐shallow dives (2% ± 3%). In the Gully, less time was spent foraging overall (5% ± 7% in short‐shallow foraging dives; 27% ± 8% in long‐deep foraging dives).

### 3.3 Three‐Dimensional Orientation and Movements

**FIGURE 6:**
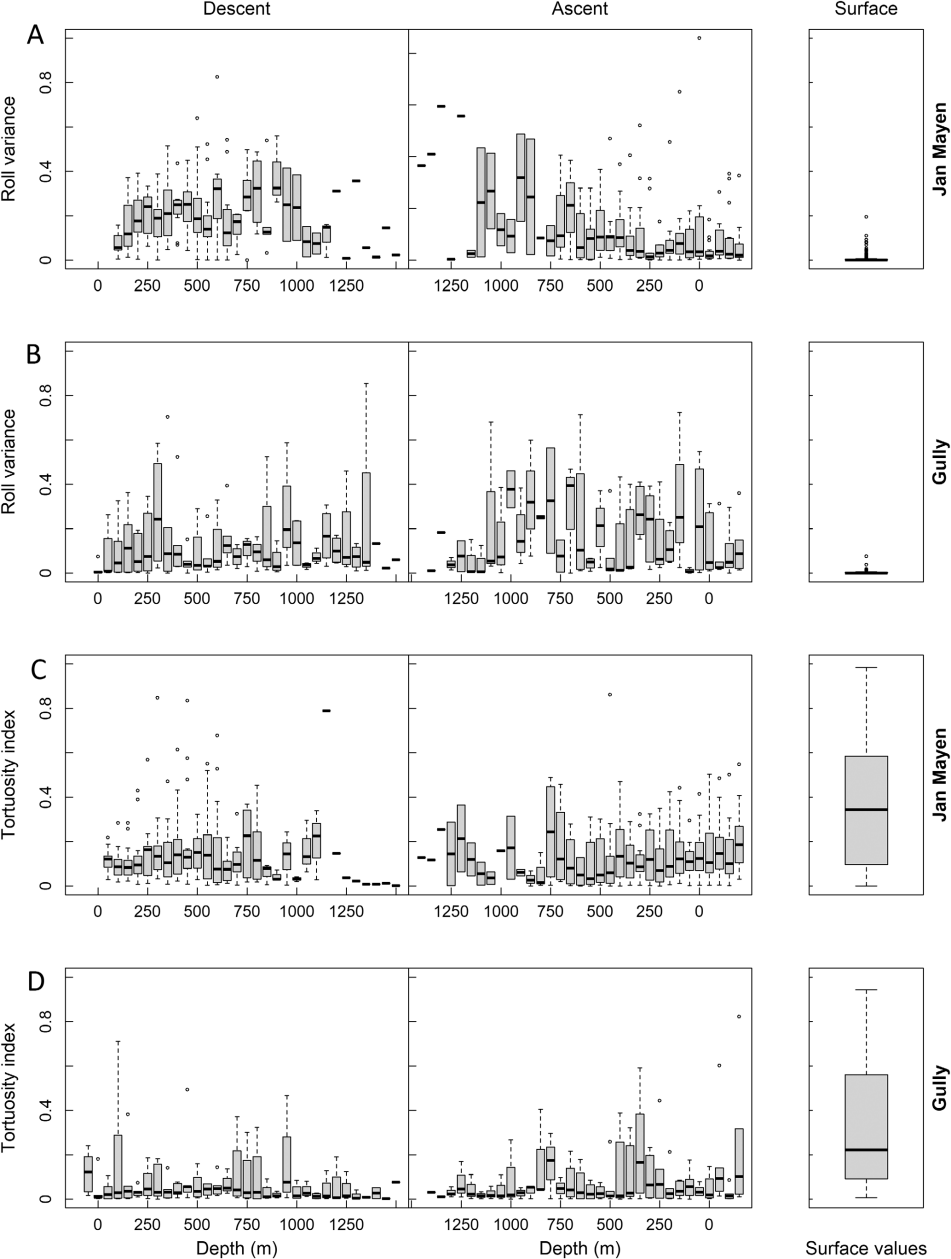
Boxplots showing individual average roll variance (A, B) and tortuosity index (C, D) in 50‐m bands during descent (left), ascent (middle) and at the surface (right) for northern bottlenose whales around Jan Mayen, Norway (A, C, *n* = 349) and the Gully, Canada (B, D, *n* = 70).

Examination of roll variance during ascent and descent suggested greater variance during initial descent in Jan Mayen, but during final ascent in the Gully (Figure 6). Heading tortuosity increased during the last 300 m of ascent to the surface in both locations but was greater in Jan Mayen. Near surface movements showed low roll variance but high and variable tortuosity (Figure 6).

**FIGURE 7:**
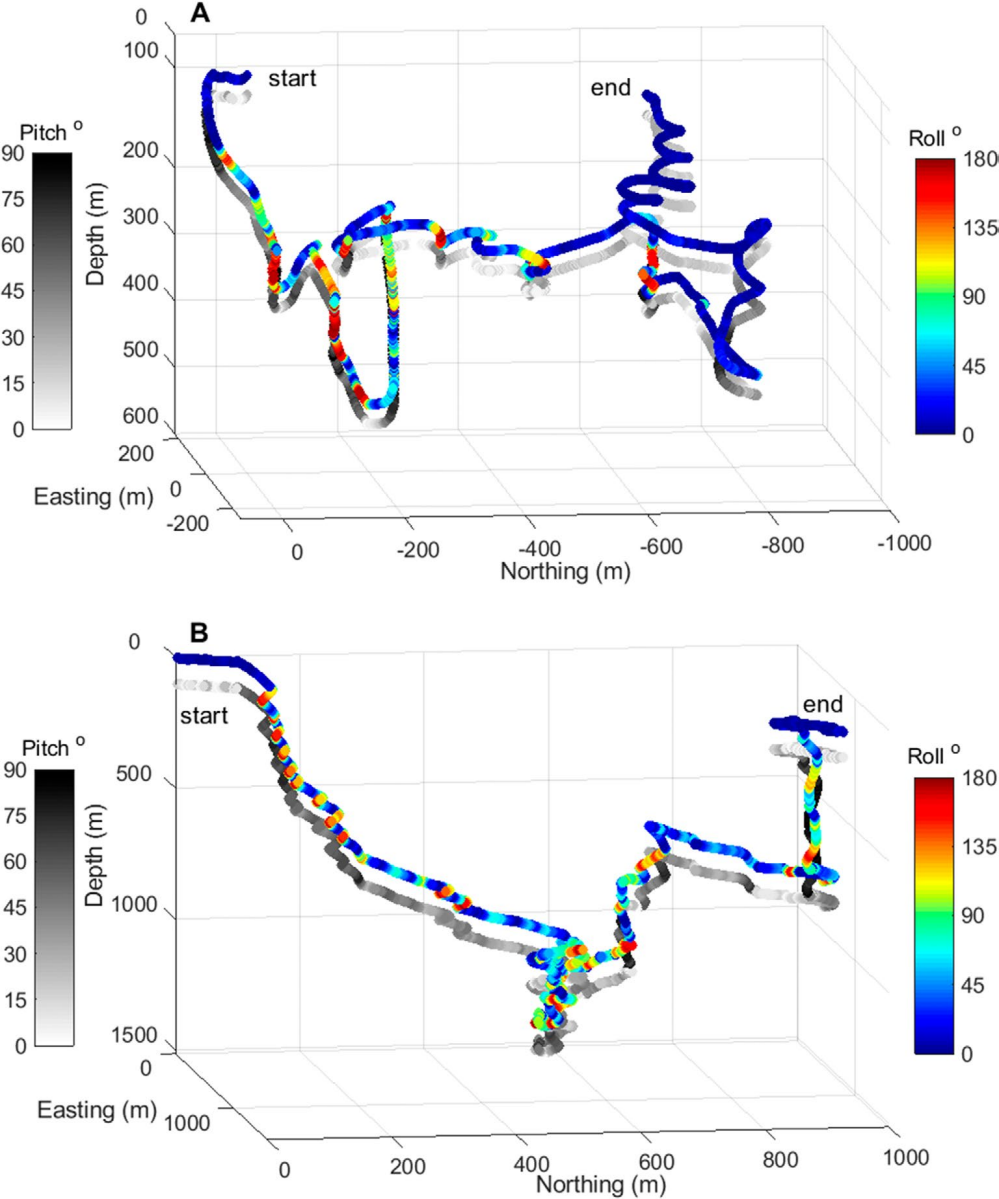
Three‐dimensional dead‐reckoned tracks coloured by roll and pitch for a single northern bottlenose whale dive in: (A) Jan Mayen (ha14_174a, pitch track offset by 30 m); (B) the Gully (11 Aug 2011, pitch track offset by 100 m). Easting and northing coordinates are relative to the whale's position at the start of the dive.

Visual inspection of dead‐reckoned tracks showed repeated patterns of gyrations (from 0° to 360°) in heading during ascent phases and in roll during descent phases. Four rotations in heading can be seen during an example ascent from 170 m to 40 m in Jan Mayen (Figure 7a), and nine continuous rotations (in roll) can be seen during an example descent from 100 to 700 m in the Gully (Figure 7b). At least two roll gyrations in the descent phase of mid‐depth and long‐deep dives were evident on 42 occasions in Jan Mayen (during 13/15 deployments) and 2 occasions in the Gully (during 2/6 deployments, Table 2). During ascent, two heading gyrations were evident on at least 32 occasions in Jan Mayen (during 11/15 deployments) and 4 occasions in the Gully (1/6 deployments, Table 2).

### 3.4 Stable Isotope Analysis

**FIGURE 8:**
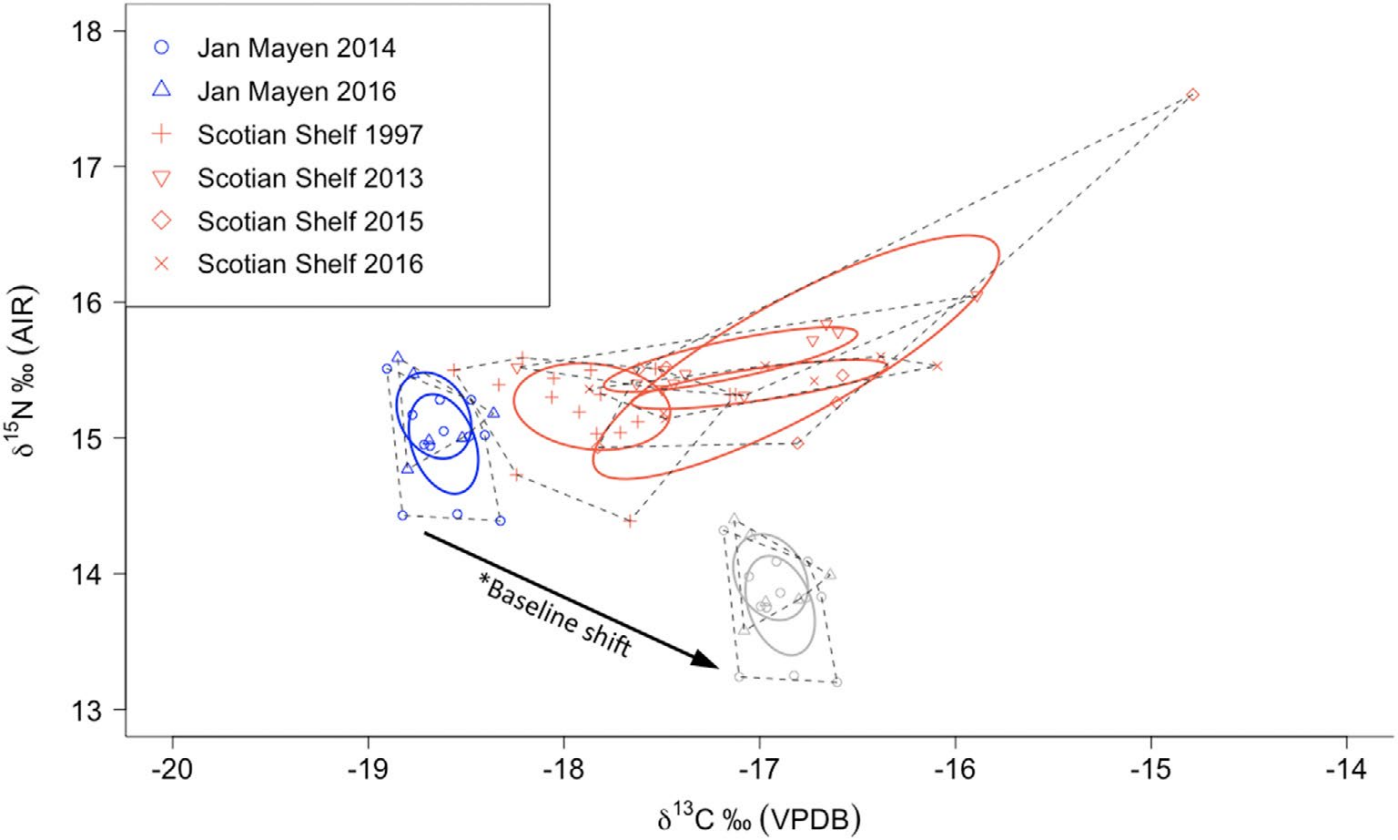
Isotopic niches determined using SIBER package for *δ* ^15^N‰ (AIR) and Suess‐corrected *δ* ^13^C‰ (VPDB) from skin tissue samples of whales from Jan Mayen and the Scotian Slope. Core isotopic niche ellipses (solid line) and total niche convex hulls (dashed lines) are shown for each sampling year and location. Arrow indicates projected shift in isotopic foraging niche of Jan Mayen whales after accounting for approximate difference in baseline zooplankton *δ* ^13^C and *δ* ^15^N values for Jan Mayen and Scotian Shelf ecoregions (+1.7‰ and −1.2‰, respectively) (Espinasse et al. 2022).

Skin samples were obtained for 18 whales in Jan Mayen (13F, 5M) and 41 whales on the Scotian Shelf (24F, 17M; Table 4). Stable isotope results showed clear differences between whales in Jan Mayen and those on the Scotian Shelf, with convex hulls showing overlaps within but not between the two locations (Figure 8). Results for *δ* ^13^C were significantly lower for Jan Mayen than for the Scotian Shelf (two‐sample *t*‐test *T* = −7.14, *p* < 0.0001). Despite substantial overlap in results, *δ* ^15^N was significantly lower for whales from Jan Mayen than for whales from the Scotian Shelf (two‐sample *t*‐test *T* = −3.25, *p* = 0.0019). One sample from 2015 Scotian Shelf had unusually high values of both *δ* ^13^C (−14.76‰) and *δ* ^15^N (+17.53‰). The difference between Jan Mayen and Scotian Shelf samples was explored without this potential outlier but remained significant (*δ* ^15^N two‐sample *t*‐test *T* = −3.75, *p* = 0.0004; Scotian Shelf mean *δ* ^15^N = +15.36‰ ± 0.23‰ AIR; *δ* ^13^C two‐sample *t*‐test *T* = −7.94, *p* < 0.0001; Scotian Shelf mean *δ* ^13^C = −17.41‰ ± 0.64‰ VPDB).

**TABLE 4:** Comparison of stable isotope mean and niche metrics for skin samples of northern bottlenose whales in Jan Mayen, Norway, versus the Gully, Canada.

Location	Sample year	*n*	Sexes	*δ* ^15^N‰ (AIR) M ± SD	*δ* ^13^C‰ (VPDB) M ± SD	*δ* ^13^C‰ (VPDB) *–* Suess corrected M ± SD	TA (‰^2^)	SEAc (‰^2^)
Jan Mayen	2014	12	8F: 4M	+14.96 ± 0.36	−18.55 ± 0.18	−18.62 ± 0.18	0.45	0.21
	2016	6	5F:1M	+15.17 ± 0.31	−18.67 ± 0.19	−18.67 ± 0.19	0.20	0.23
	**All**	**18**	13F:5M	**+15.03** ± **0.35**	**−18.59 ± 0.18**	**−18.63 ± 0.18**		
Scotian Shelf	1997	16	12F:4M	+15.23 ± 0.32	−17.38 ± 0.40	−17.86 ± 0.40	1.03	0.42
	2013	7	2F:5M	+15.39 ± 0.18	−16.91 ± 0.65	−17.00 ± 0.65	0.33	0.29
	2015	7	4F:3M	+15.60 ± 0.89	−16.79 ± 1.03	−16.81 ± 1.03	1.89	1.85
	2016	11	5F:6M	+15.58 ± 0.24	−17.15 ± 0.64	−17.15 ± 0.64	0.57	0.32
	**All**	**41**	23F:18M	**+15.41 ± 0.45**	**−17.14 ± 0.66**	**−17.34 ± 0.75**		

*Note:* Samples are pooled by year in which the biopsy was collected. Results based on all samples for each location are shown in bold. Total Area (TA) of the convex hulls encompass 100% of the data points per species. Ellipses encompass 40% of the data (per region and collection year) and provide core isotopic niche (standard ellipse area corrected for small sample size [SEAc]). Isotopic niche metrics presented in ‰^2^.

## 4 Discussion

This study provides evidence of geographic differences in dive types, foraging depths, time budgets and stable isotope values between the north‐eastern and north‐western Atlantic populations of northern bottlenose whales. In Jan Mayen, whales consistently foraged over a wider depth range with a substantial number of mid‐depth dives, while Gully whales primarily foraged at deep depths. Jan Mayen whales spent more time foraging and less time resting near the surface than whales in the Gully.

Carbon and nitrogen isotopes suggested potentially lower trophic level foraging in Jan Mayen than the Gully (Figure 8). However, the baseline isoscape correction applied (Espinasse et al. 2022) should be treated with some caution as Jan Mayen sits at the border of two ecoregions and the alternative region, while giving the same correction for *δ* ^13^C, would give a +0.4‰ correction rather than a −1.2‰ correction for *δ* ^15^N (Espinasse et al. 2022).

### 4.1 Deep/Mid‐Water Foraging

Long‐deep and short‐shallow dives have previously been observed in the Gully and Jan Mayen (Hooker and Baird 1999; Miller et al. 2015). This study additionally showed the presence of mid‐depth dives (300–700 m) in Jan Mayen that were rarely present in the Gully.

Beaked whale foraging dives are usually identified using click presence to indicate periods of prey searching and prey capture (Madsen et al. 2013; Miller et al. 2015). However, this is feasible only when hydrophone data is available. Roll was therefore used as an alternative indicator for foraging, and was examined against click presence when both datasets were available. In fact neither measure is necessarily an absolute indicator of foraging (i.e., there may be silent foraging dives and there may be non‐rolling foraging dives). However, congruence was high between these indicators, as has been found for other studies and species (Miller, Johnson, and Tyack 2004; Madsen et al. 2005; Stimpert et al. 2014; Sweeney et al. 2022). Foraging dives identified by high roll variance in this study showed characteristics consistent with active foraging behaviour in deep‐diving odontocetes: a high proportion of time spent during the bottom phase, high jerk CV and heading variance (Miller, Johnson, and Tyack 2004; Irvine et al. 2017). Beaked whales often cease echolocation in response to acoustic disturbance (Miller et al. 2015), but the five deep non‐clicking dives observed here (Figure A2) did not appear to be associated with disturbance events (such as time since tag placement).

Long‐deep dives have previously been suggested to descend near to the sea floor in the Gully (Hooker and Baird 1999). Dives in Jan Mayen were less deep but might also have been near the sea floor (Figure 1). Faster descent and ascent rates in the Gully (Table 2) were possibly due to increased vertical transit rates with greater body density deviation from neutral buoyancy (Miller et al. 2012, 2016). However, the major difference between diving in the Gully and Jan Mayen was the prevalence of mid‐depth foraging (300–700 m) in Jan Mayen, but the near absence of this in the Gully.

Environmental factors driving heterogeneity in food resources are typically considered the main driver of inter‐population variation in foraging behaviour (Foster 1999). On both sides of the North Atlantic, northern bottlenose whales predate on deep‐water fish and cephalopods, in particular *Gonatus* squid (Whitehead and Hooker 2012). Fatty acid signatures of *Gonatus* and bottlenose whale skin samples suggested that northern bottlenose whales may preferentially target adult female *Gonatus* (Hooker et al. 2001). During their 2‐year life cycle, *Gonatus* squid undergo ontogenetic descent, with larger, more mature squid found at greater depths, although juveniles and active swimming adult males can be found as shallow as 200 m (Golikov et al. 2018). The steep topography and depth of canyon features in both the Gully and around Jan Mayen may drive oceanographic processes such as downwelling (Moors‐Murphy 2014). Adult female *Gonatus* undergo degenerative loss of musculature and are aggregated by downwelling (Gardiner and Dick 2010), likely rendering them easy to catch prey. The long‐deep foraging dives and lack of mid‐depth foraging in the Gully may therefore arise from whales feeding primarily on aggregations of adult female squid at great depths. Low heading variance, indicating few orientation changes in the bottom phase (Figure 4), is consistent with feeding on less mobile prey.

In addition to squid, the diet of whaled animals in Labrador and Iceland has also contained fish (Benjaminsen and Christensen 1979), and whales on the Grand Banks have been observed to scavenge from fishing vessels (Oyarbide et al. 2023). Active mid‐water foraging dives in Jan Mayen may therefore reflect predation on mid‐water fish or juvenile squid, which would manifest in lower *δ* ^13^C and *δ* ^15^N‰ than whales consuming predominantly high trophic level adult squid. These dives had particularly high jerk CV (acceleration) and heading variance (changes in orientation) in the bottom phase (Figure 4), which are also consistent with foraging on more mobile prey. In contrast, long‐deep foraging dives had lower jerk CV and heading variance (Figure 4) which may indicate that, as in the Gully, Jan Mayen whales target less mobile prey at greater depths.

### 4.2 Shallow Dives

In both locations, some short‐shallow dives appeared to contain foraging‐type movements (Figure 4). Some of these high‐roll variance dives nevertheless had no echolocation clicks. It is possible that some foraging might rely on visual cues, rather than echolocation clicks (Madsen et al. 2013), or that these dives might have another active but silent behavioural function. Short‐shallow dives in the Gully had lower jerk CV values than Jan Mayen (Figure 4). However, the single suction‐cup attachment used for four Gully tag deployments may cloud this comparison, as its less rigid attachment will be less sensitive to jerk movements. In addition, the slight difference in sampling rates (50 Hz for Dtag and 32 Hz for LL tag) will have affected jerk calculations (Ydesen et al. 2014), although jerk CV is much less sensitive to differences in sampling rates than jerk itself. Accelerometer clipping level (here ±2 *g* or 3 *g*) is another concern (Ydesen et al. 2014), since clipping during prey‐capture attempts can also result in an underestimation of the acceleration exhibited during foraging. However, low heading variance and low percentage of time in the bottom phase (Figure 4) are also inconsistent with foraging (Tennessen et al. 2019). These Gully shallow dives might therefore have another function, e.g., resting, digestion, social or anti‐predator (Tyack et al. 2006). These are not mutually exclusive, and would require independent assessment (e.g., camera tag) to better elucidate function.

### 4.3 Residency and Migration

Whales around Jan Mayen spent significantly more time in foraging dives (47% total time) and almost twice the proportion of time in the bottom phase of foraging dives (23% total time, Table 3). Gully whales spent more of their time near the surface, with low roll variance suggesting that foraging likely did not occur. Such contrasts in time budgeted to foraging suggest inter‐population differences in energetic demands or prey energy content. Unlike Jan Mayen whales, which are known to migrate south (Miller et al. 2016; Haas et al. 2026), several lines of evidence (photo‐identification, acoustic detection, morphology and genetic analysis) indicate that bottlenose whales on the Scotian Shelf form a distinct resident population (Whitehead et al. 1997; Gowans, Whitehead, et al. 2000; de Greef et al. 2022; Feyrer, Stanistreet, Gomez, et al. 2024). Not subject to the high energetic demands required for long‐distance migrations, resident populations across animal taxa consistently spend less time foraging than migrant populations (Robinson and Merrill 2013).

Other contextual factors such as diel changes in either prey or predation risk could also affect differences in time budgets. For example, vertical migration of prey and/or a reduced risk of visual detection by shallow‐diving predators at night are thought to cause other beaked whales to spend a higher proportion of time near the surface at night (Baird et al. 2008). However, all Jan Mayen deployments (collected at high latitude during summer) were obtained under daylight conditions, and the one overnight Gully deployment (06Sep2013, Table 2) spent a relatively low percentage of time near the surface (Figure 5), suggesting that diel changes had little effect on time‐budget differences between the locations.

### 4.4 Orientation and Three‐Dimensional Movements

Rolling during descent and enigmatic heading gyrations in ascent were more apparent in Jan Mayen although they also occurred in the Gully (Table 2). Rolling in descent could enhance prey searching as northern bottlenose whales have a highly directional sonar beam (Wahlberg 2011) so rolling would widen the ensonified search area. Heading gyrations have also been observed for other species during ascent from consecutive deep/medium dives (Narazaki et al. 2021). Such movements, potentially enhancing visual or acoustic searching, could be due to the need to regroup prior to surfacing (Aguilar de Soto et al. 2020).

Northern bottlenose whales glide at the end of ascent due to expanding lung gases (Miller et al. 2016). Circular movements at reduced pitch angles might help counter the buoyancy effects of expanding lung gases, allowing slower ascent with less horizontal displacement. Using low pitch gyrations to manage buoyancy forces could further explain why there were more instances of gyrations in Jan Mayen where whales had lower body density and more positive net buoyancy (Miller et al. 2016).

### 4.5 Implications

This study highlights the risks of assuming a local population‐level dataset is ecologically representative of the species across a global context. Northern bottlenose whales have been managed as one stock by the IWC (IWC 1977), but this study showed consistent and multi‐faceted differences between two geographically separated populations of whales.

Increasing anthropogenic pressures in the Arctic are a particular current cause for concern for the North Atlantic (Post et al. 2013; Reeves et al. 2014; Ladegaard et al. 2021). Short‐term disturbance from anthropogenic (sonar) noise appears to cause bottlenose whales to halt foraging behaviour (Miller et al. 2015; Wensveen et al. 2019; Stanistreet et al. 2022). Whales in Jan Mayen, which spend 50% more time foraging, are therefore likely to be particularly impacted by noise disturbance. Lost foraging opportunities may have a greater effect for Jan Mayen whales given their need for additional lipid stores for migratory behaviour. Although the Scotian Shelf population in the Gully does not appear to migrate, they might nevertheless be more prone to suffering fitness consequences of disrupted foraging due to their lower blubber reserves. Modelling the population consequences for these high‐intensity versus low‐intensity foragers are therefore likely to result in very different impacts caused by the same industrial activity (Pirotta et al. 2018). Differences in behaviour in terms of time spent resting and time spent foraging will also change both acoustic and visual detectability of animals and potentially therefore impact population estimates (Barlow et al. 2025). Such inter‐population variation in behaviour requires increased consideration in future development of conservation strategies.

## Author Contributions

**Sascha K. Hooker:** conceptualization (equal), formal analysis (equal), methodology (equal), project administration (equal), supervision (equal), writing – original draft (lead), writing – review and editing (equal). **Eilidh Siegal:** conceptualization (equal), data curation (lead), formal analysis (lead), investigation (equal), methodology (equal), project administration (equal), writing – original draft (equal), writing – review and editing (equal). **Tessa Plint:** data curation (equal), formal analysis (lead), writing – original draft (equal), writing – review and editing (equal). **Laura J. Feyrer:** funding acquisition (equal), investigation (equal). **Tomoko Narazaki:** investigation (equal). **Kagari Aoki:** investigation (equal). **Matt Bivins:** investigation (equal). **Patrick J. O. Miller:** conceptualization (equal), formal analysis (equal), funding acquisition (lead), investigation (equal), methodology (equal), project administration (lead), supervision (equal), writing – original draft (equal), writing – review and editing (equal).

## Funding

This work was supported by the Direction Générale de l'Armement (DGA), France; Office of Naval Research, United States; Strategic Environmental Research and Development Program, United States RC‐2337.

## Conflicts of Interest

The authors declare no conflicts of interest.

## Data Availability

Dryad digital repository reviewer URL: https://doi.org/10.5061/dryad.dbrv15fcd.

## Appendix 1 Dive Classification

Comparison of the study dataset with downsampled data for Jan Mayen, or with inclusion of additional data for the Gully suggested that sample size had not unduly affected the number of dive type clusters identified.

## Appendix 2 Identification of Foraging Dives

Using the presence or absence of regular clicks as a foraging indicator (available for the Jan Mayen dataset), an optimal roll variance threshold was estimated, above which high roll variance dives were considered ‘foraging dives’ and below which low roll variance dives were considered ‘non‐foraging dives’. Over a range of roll variance thresholds (from 0 to 1 at increments of 0.01), the number of mismatched dives was estimated as: (# dives with clicking < threshold) + (# dives without clicking > threshold). The optimal roll variance threshold was that which minimised the number of mismatched dives (dives considered different by acoustic versus roll variance).
